# MYC-induced reprogramming of glutamine catabolism supports optimal virus replication

**DOI:** 10.1038/ncomms9873

**Published:** 2015-11-12

**Authors:** Minh Thai, Shivani K. Thaker, Jun Feng, Yushen Du, Hailiang Hu, Ting Ting Wu, Thomas G. Graeber, Daniel Braas, Heather R. Christofk

**Affiliations:** 1 Department of Molecular and Medical Pharmacology, David Geffen School of Medicine, University of California, Los Angeles, 90095 California, USA; 2 Department of Pathology and Laboratory Medicine, David Geffen School of Medicine, UCLA, Los Angeles, 90095 California, USA; 3 Jonsson Comprehensive Cancer Center, David Geffen School of Medicine at UCLA, Los Angeles, 90095 California, USA; 4 Crump Institute for Molecular Imaging, David Geffen School of Medicine, University of California, Los Angeles, 90095 Californa, USA; 5 UCLA Metabolomics Center, Los Angeles, 90095 California, USA; 6 Eli and Edythe Broad Center of Regenerative Medicine and Stem Cell Research, University of California, Los Angeles, 90095 California, USA

## Abstract

Viruses rewire host cell glucose and glutamine metabolism to meet the bioenergetic and biosynthetic demands of viral propagation. However, the mechanism by which viruses reprogram glutamine metabolism and the metabolic fate of glutamine during adenovirus infection have remained elusive. Here, we show MYC activation is necessary for adenovirus-induced upregulation of host cell glutamine utilization and increased expression of glutamine transporters and glutamine catabolism enzymes. Adenovirus-induced MYC activation promotes increased glutamine uptake, increased use of glutamine in reductive carboxylation and increased use of glutamine in generating hexosamine pathway intermediates and specific amino acids. We identify glutaminase (GLS) as a critical enzyme for optimal adenovirus replication and demonstrate that GLS inhibition decreases replication of adenovirus, herpes simplex virus 1 and influenza A in cultured primary cells. Our findings show that adenovirus-induced reprogramming of glutamine metabolism through MYC activation promotes optimal progeny virion generation, and suggest that GLS inhibitors may be useful therapeutically to reduce replication of diverse viruses.

## Abstract (web summary)

Viruses can reprogram glutamine metabolism of host cells to support bioenergetics demands of viral replication. Here the authors show that adenoviral infection leads to enhanced glutamine metabolism through virus-mediated activation of MYC, which is required for optimal progeny virion generation.

## Graphical abstract

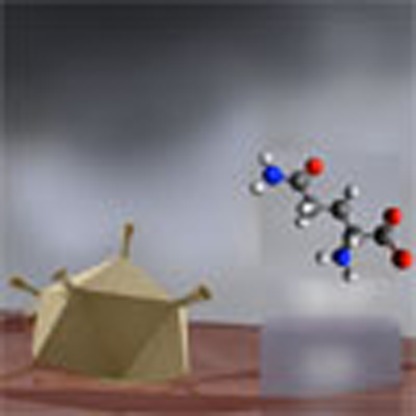

Glutamine is a critical nutrient for cellular biosynthetic processes. Many cancer cells rely on increased glutamine metabolism for growth and use glutamine as a source of carbon and nitrogen atoms for synthesis of lipids, nucleotides and certain amino acids^1^,^2^, as well as a cellular exchange factor for uptake of essential amino acids^3^. Similar to cancer cells, viruses require increased anabolic metabolism to support proliferation^4^,^5^,^6^, and therefore may also depend on increased glutamine metabolism for replication. Consistently, glutamine deprivation decreases human cytomegalovirus (HCMV) replication in human foreskin fibroblasts (HFF)—HCMV-infected cells reportedly use glutamine to fuel ATP production and anaplerosis in the tricarboxylic acid (TCA) cycle^7^. However, whether glutamine uptake is important for replication of other viruses, how glutamine metabolism is regulated during viral infection, and whether pharmacological inhibition of glutamine metabolism can affect replication of diverse viruses have remained unknown.

We recently reported that the gene product of adenovirus E4ORF1 enhances host cell anabolic glucose metabolism during virus replication through MYC activation^6^. E4ORF1 binds to nuclear MYC and enhances MYC-induced transcription of specific glycolytic target genes. A D68A point mutation in E4ORF1 abrogates binding to and activation of MYC, and a mutant adenovirus 5 containing the D68A point mutation in E4ORF1 (AD ORF1 D68A) that is deficient in MYC activation exhibits impaired reprogramming of glucose metabolism and virus replication in primary lung epithelial cells. Since MYC enhances glutamine metabolism in cancer cells in part through promoting increased expression of glutaminase (GLS)^8^ and the glutamine transporters ASCT2 and SN2 (ref. 9), we hypothesized that E4ORF1-induced MYC activation may enhance glutamine metabolism during adenovirus infection through modulation of GLS and glutamine transporter levels. Here, we characterize changes in glutamine metabolism in primary lung epithelial cells after infection with a wild-type (WT) strain of adenovirus 5 (AD). Additionally, we compare AD WT infection to AD ORF1 D68A infection to assess effects of adenovirus-mediated MYC activation on regulation of glutamine metabolism during infection.

## Results

### MYC regulates glutamine uptake upon adenovirus infection

**Figure 1:**
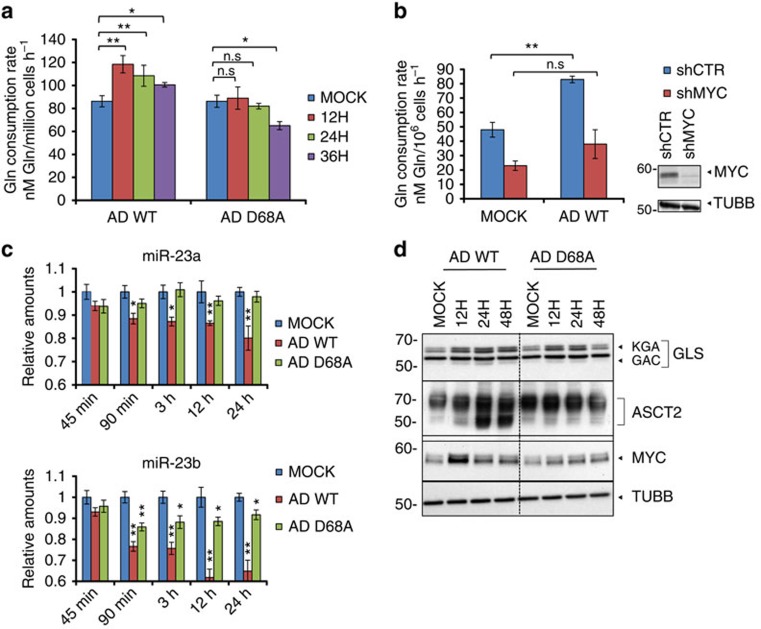
MYC regulates glutamine consumption during adenovirus infection. (**a**) NHBE cells were either mock infected, infected with AD WT (wild-type strain of adenovirus 5), or infected with AD ORF1 D68A, and cellular glutamine consumption rates were measured at the indicated time points. (**b**) (left) Glutamine consumption rates of NHBE cells stably expressing scrambled shRNA (shCTR, blue bars) or MYC shRNA (shMYC, red bars) 24 h post mock infection or infection with AD WT (left). (right) Immunoblotting depicting MYC levels in NHBE cells stably expressing scrambled shRNA (shCTR) or MYC shRNA (shMYC). Tubulin antibody (TUBB) was used to control for loading. (**c**) Relative miR-23a and mir-23b levels in NHBE cells mock infected, infected with AD WT, or infected with AD ORF1 D68A at the indicated time points post infection. (**d**) Immunoblotting of lysates from NHBE cells infected with AD WT or AD ORF1 D68A at the indicated times post infection. Whole cell lysates were probed with antibodies towards GLS (glutaminase) and ASCT2 (sodium-dependent neutral amino acid transporter type 2). KGA is the full-length GLS and GAC is the shortened splice variant^10^. Nuclear lysates were probed with an antibody towards MYC. Tubulin antibody (TUBB) was used to control for loading in the whole cell lysates. For (**a**–**c**), error bars denote s.d. (*n*=3), *P<0.05; **P<0.01. Student's *t*-test.

To determine whether adenovirus infection alters host cell glutamine metabolism, we measured glutamine consumption rates of primary normal human bronchial epithelial cells (NHBE) at multiple time points after AD WT infection. AD WT infection causes increased glutamine consumption rates at early time points post infection (Fig. 1a). Our data suggest that the adenovirus-induced increase in glutamine consumption is MYC-dependent since cells infected with a mutant adenovirus deficient in MYC activation, AD ORF1 D68A^6^, do not exhibit increased glutamine consumption rates at early time points post infection (Fig. 1a). Furthermore, short hairpin RNA (shRNA)-mediated knockdown of MYC impairs the ability of AD WT to enhance glutamine consumption in NHBE cells (Fig. 1b, Supplementary Fig. 5a).

Since MYC regulates cancer cell glutamine metabolism in part through suppression of miR-23a and miR-23b, which are microRNAs that target the GLS 3′-UTR and thereby decrease GLS expression^8^, we tested whether MYC modulates miR-23a/b and GLS levels during adenovirus infection of NHBE cells. We found that AD WT infection causes reduced expression of both miR-23a and miR-23b as early as 90 min post infection (Fig. 1c). AD ORF1 D68A infection, however, has no effect on miR-23a levels, and decreases miR-23b levels to a lesser extent than AD WT infection of NHBE cells (Fig. 1c). Consistently, AD WT infection leads to a larger increase in GLS protein levels, including GLS splice variants KGA (full-length GLS) and GAC (shortened splice variant)^10^, than AD ORF1 D68A infection of NHBE cells (Fig. 1d; Supplementary Fig. 5b). Since MYC also drives glutamine metabolism by activating expression of genes involved in glutamine uptake, including *SLC7A5* and *SLC1A5*, which encode the glutamine transporters LAT1 and ASCT2, respectively^11^, we measured expression of these transporters during adenovirus infection. We found that AD WT, but not AD ORF1 D68A, infection increases transcription of LAT1 and ASCT2 in MCF10A cells (Supplementary Fig. 1a). Additionally, AD WT, but not AD ORF1 D68A, modestly increases ASCT2 protein levels in NHBE cells (Fig. 1d; Supplementary Fig. 5b). Together, our data support MYC activation-dependent regulation of glutamine metabolism during adenovirus infection through repression of miR-23 leading to upregulation of GLS, as well as MYC-dependent upregulation of the glutamine transporters, ASCT2 and LAT1.

### Adenovirus infection alters host cell glutamine utilization

**Figure 2:**
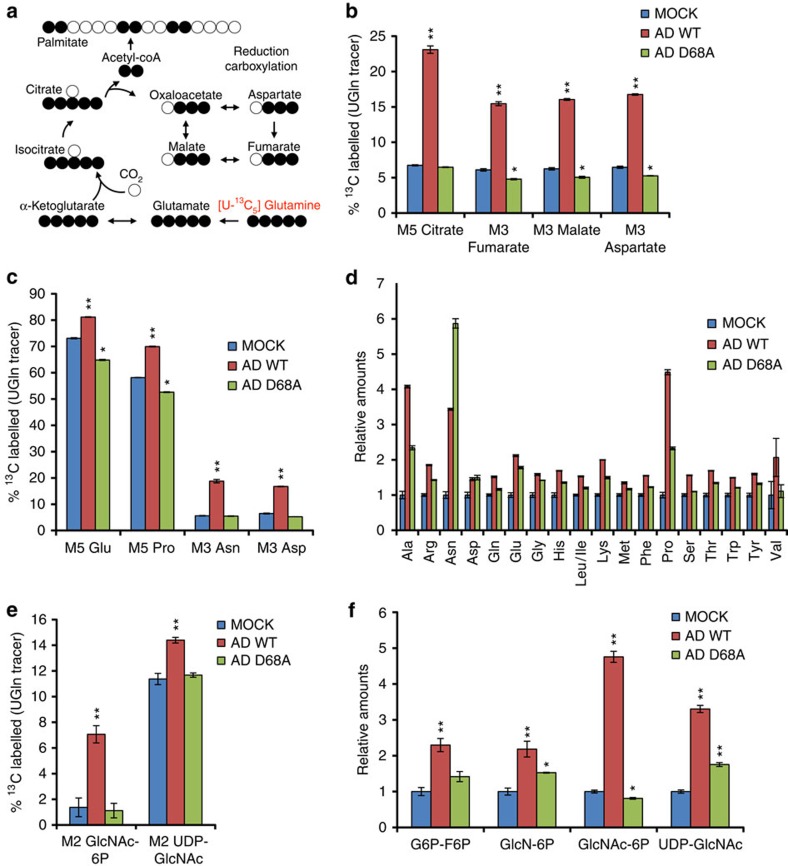
Adenovirus infection alters host cell glutamine utilization in a MYC activation-dependent manner. NHBE cells were labelled with U-^13^C_5_-glutamine and mock infected or infected with AD WT or AD ORF1 D68A at an MOI of 1. 24 h post infection, intracellular metabolites were extracted and analysed by LC-MS/MS. (**a**) Schematic tracing the fate of ^13^C atoms from U-^13^C_5_-glutamine in reductive carboxylation. (**b**) Percentage of ^13^C-labelled isotopomers of intermediates in reductive carboxylation. (**c**) Percentage of ^13^C-labelled isotopomers of amino acids. (**d**) Relative levels of non-essential and essential amino acids. (**e**) Percentage of ^13^C-labelled M2 isotopomers of intermediates in the hexosamine biosynthesis pathway. (**f**) Relative levels of hexosamine biosynthesis pathway intermediates. For (**b**–**f**), error bars denote s.d. (*n*=3), *P<0.05; **P<0.01. Student's *t*-test.

To characterize MYC-induced changes in glutamine metabolism during adenovirus infection, NHBE cells were labelled with U-^13^C_5_-glutamine and mock infected or infected at an multiplicity of infection (MOI) of 1 with AD WT or AD ORF1 D68A. Twenty-four post labelling and infection, metabolites were extracted and analysed by liquid chromatography tandem mass spectrometry (LC-MS/MS). AD WT infection, but not AD ORF1 D68A infection (MYC activation-deficient), increases the percentage of intracellular M5 isotopologue-labelled (five carbon-labelled) glutamate relative to mock-infected cells, consistent with a MYC activation-dependent increase in GLS-catalysed conversion of glutamine to glutamate during adenovirus infection (Supplementary Fig. 1b,c). AD WT infection, but not AD ORF1 D68A infection, also increases the percentage of M5-labelled alpha-ketoglutarate (Supplementary Fig. 1b,c). Typically, activity of the oxidative citric acid cycle results in elevated levels of M4-labelled TCA cycle intermediates in cells labelled with U-^13^C_5_-glutamine (Supplementary Fig. 1b). However, AD WT infection of NHBE cells causes a decrease in M4-labelled fumarate, malate, aspartate and citrate (Supplementary Fig. 1c,d). Instead, AD WT infection, but not AD ORF1 D68A infection, increases the levels of M5-labelled citrate, M3-labelled fumarate, M3-labelled malate and M3-labelled aspartate (Fig. 2a,b, Supplementary Fig. 1d). This labelling pattern of TCA cycle intermediates from U-^13^C_5_-glutamine metabolism suggests a MYC-dependent increase in reductive carboxylation upon adenovirus infection^12^.

To determine whether transcription of enzymes involved in reductive carboxylation is increased upon adenovirus infection, we performed real-time quantitative PCR on several enzymes that have been shown to mediate reductive glutamine metabolism^12^,^13^,^14^. We detect increased transcript levels of isocitrate dehydrogenases 1 and 2 (*IDH1* and *IDH2*), nicotinamide nucleotide transhydrogenase (*NNT*), pyruvate dehydrogenase kinase isoenzyme 1 (*PDK1*) and glutamic-oxaloacetic transaminase 2 (*GOT2*) in host cells at 8 h post infection with AD WT, but not in cells infected with AD ORF1 D68A (Supplementary Fig. 1e). These data support a model whereby adenovirus-induced MYC activation increases host cell reductive glutamine metabolism through transcriptional upregulation of enzymes involved in reductive carboxylation during infection (Supplementary Fig. 2).

Since glutamine is used by cells to generate non-essential amino acids and import essential and non-essential amino acids, we assessed the impact of adenovirus infection on abundance and labelling patterns of intracellular amino acid pools in NHBE cells labelled with U-^13^C_5_-glutamine and infected with AD WT or AD ORF1 D68A. In addition to the previously described increased percentage of intracellular M5-labelled glutamate (Fig. 2c and Supplementary Fig. 1c), AD WT infection, but not AD ORF1 D68A infection, also causes elevated M5-labelled proline, M3-labelled asparagine and M3-labelled aspartate (Fig. 2c). Notably, AD WT infection also causes increased intracellular levels of essential and non-essential amino acids in NHBE cells more so than AD ORF1 D68A infection, with the exception of asparagine (Fig. 2d). The elevated asparagine levels in AD ORF1 D68A-infected cells may reflect differential regulation or use of asparagine versus the other amino acids in adenovirus-infected cells. Consistent with the increased intracellular levels of most amino acids, we found that transcript levels of *ASCT2* and *LAT1*, and protein levels of ASCT2, transporters that utilize glutamine as an exchange factor for amino acid uptake^3^, are significantly elevated in AD WT-infected cells, but not AD ORF1 D68A infected cells (Fig. 1d and Supplementary Fig. 1a), suggesting MYC-dependent regulation of glutamine exchange for amino acid uptake.

Since glutamine can contribute to the hexosamine biosynthesis pathway through providing a nitrogen atom for conversion of fructose-6-phosphate to glucosamine-6-phosphate, and through providing carbon atoms for conversion of glucosamine-6-phosphate to GlcNAc-6-phosphate through production of acetyl-CoA from citrate, we examined the levels and labelling pattern of hexosamine pathway intermediates in NHBE cells labelled with U-^13^C_5_-glutamine and infected with AD WT or AD ORF1 D68A using LC-MS/MS. AD WT infection, but not AD ORF1 D68A infection, causes increased levels of M2-labelled GlcNAc-6P/GlcNAc-1P and M2-labelled UDP-GlcNAc (Fig. 2e). AD WT infection also causes increased intracellular pool sizes of hexosamine pathway intermediates (Fig. 2f), and increases in transcript levels of hexosamine biosynthesis pathway enzymes including hexokinase 2 (HK2)^6^, glutamine fructose-6-phosphate amidotransferase 1 (GFAT1), encoded by the *GFPT1* gene and *N*-acetylglucosamine kinase (NAGK) significantly more than AD ORF1 D68A infection (Supplementary Fig. 3a). These findings suggest MYC-dependent changes in hexosamine biosynthesis during adenovirus infection.

### GLS activity promotes optimal adenovirus replication

**Figure 3:**
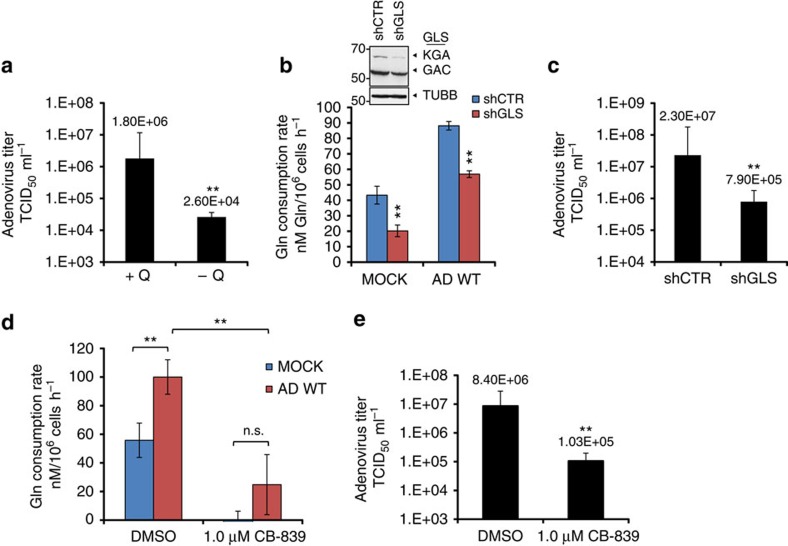
Glutamine consumption and GLS activity are important for optimal adenovirus replication. (**a**) NHBE cells were infected with AD WT at an MOI of 1 in the presence (+ Q) or absence (−Q) of 2.5 mM glutamine. At 24 h post infection, progeny virus was harvested and infectious virus titer was measured using a TCID_50_ end point dilution plaque assay. (**b**) (bottom) Glutamine consumption rates of NHBE cells stably expressing scrambled shRNA (shCTR, blue bars) or GLS shRNA (shGLS, red bars) 24 h post mock infection or infection with AD WT (bottom). (top) Immunoblotting depicting levels of GLS splice isoforms KGA and GAC in NHBE cells stably expressing scrambled shRNA (shCTR) or GLS shRNA (shGLS). (**c**) Progeny virus was collected from NHBE cells stably expressing shCTRL or shGLS 24 h post infection with AD WT, and adenovirus titres were determined as in (**a**). (**d**) NHBE cells were treated with 1.0 μM CB-839 or DMSO, and either mock-infected or infected with AD WT. Glutamine consumption rates were measured 24 h post treatment and infection. (**e**) Progeny virus was harvested from NHBE cells treated with DMSO or 1.0 μM CB-839 24 h post infection with AD WT, and adenovirus titers were determined as described in (**a**). For (**a**–**e**), error bars denote s.d. (*n*=3), **P<0.01. Student's *t*-test.

Because we observed increased glutamine utilization during adenovirus infection, we hypothesized that glutamine consumption may be essential for optimal adenovirus replication. To test this hypothesis, we assessed the impact of exogenous glutamine deprivation on adenovirus replication in primary NHBE cells. As shown in Fig. 3a, AD WT replication in the absence of glutamine was reduced 60-fold compared with replication in the presence of 2.5 mM glutamine. Since we observed increased GLS activity in AD WT-infected cells, as judged by increased conversion of ^13^C_5_-glutamine to ^13^C_5_-glutamate (Fig. 2c), we hypothesized that GLS activity may be important for adenovirus replication. To test this hypothesis, we first diminished GLS activity in adenovirus-infected primary NHBE cells by shRNA-mediated knockdown of GLS (Fig. 3b; Supplementary Fig. 5c). GLS knockdown reduces the glutamine consumption rates of mock-infected and AD WT-infected cells (Fig. 3b; Supplementary Fig. 5c), and reduces AD WT replication 25-fold relative to AD WT replication in cells expressing a scrambled shRNA control (Fig. 3c). These data suggest that glutamine consumption and conversion of glutamine to glutamate through GLS activity contribute to optimal adenovirus replication in primary NHBE cells.

Since GLS knockdown decreased adenovirus replication, we hypothesized that pharmacological inhibition of GLS may also limit progeny virion generation. Previous studies in vaccinia virus (VACV), a member of the Poxviridae family, showed that treatment of VACV-infected cells with bis-2-(5-phenylacetamido-1,3,4-thiadiazol-2-yl)ethyl sulfide (BPTES), a small-molecule GLS inhibitor, decreased viral yields in a manner similar to cells deprived of glutamine^15^. However, clinical use of BPTES is limited because of moderate potency, poor metabolic stability and low solubility^16^. Another small-molecule GLS inhibitor, CB-839, is currently undergoing phase I evaluation for treatment of patients with solid tumours and haematological malignancies, and unlike BPTES, is orally bioavailable with low nanomolar potency against both splice isoforms of GLS. CB-839 has previously been shown to decrease proliferation of triple-negative breast cancer cell lines and xenograft models, consistent with stronger reliance of these cells on extracellular glutamine for growth compared with other breast cancer cell lines^16^. Therefore we tested the impact of CB-839 on glutamine consumption rate and adenovirus replication in NHBE cells. As shown in Fig. 3d, treatment of NHBE cells with 1.0 μM CB839 dramatically reduces cellular glutamine consumption rate and blocks the increased glutamine consumption rate conferred by adenovirus infection. Importantly, CB-839 treatment significantly decreases AD WT replication by 80-fold relative to AD WT replication in dimethylsulfoxide (DMSO)-treated cells (Fig. 3e). These data suggest that CB-839 effectively reduces adenovirus replication in primary NHBE cells.

### GLS inhibition limits HSV-1 and influenza A replication

Since glutamine is an important nutrient for biosynthesis, and increased glutamine consumption and GLS activity promote optimal adenovirus replication, we hypothesized that glutamine uptake and GLS activity may be important for replication of additional clinically relevant viruses. Herpes simplex virus 1 (HSV-1) is a highly contagious enveloped double-stranded DNA virus that causes recurrent disease worldwide, most commonly leading to oral lesions, or ‘cold sores', as well as ocular and neurological manifestations in infected individuals. Although the guanosine analogue acyclovir and related compounds constitute first-line treatment against HSV-1 infection, additional combination therapeutics are needed to treat drug-resistant infections and improve efficacy, especially in high-risk patients^17^. Influenza A is an enveloped, segmented, single-stranded RNA virus that causes acute respiratory illness and fatality in severe cases. New strains of influenza A can emerge to infect humans, which sometimes cause pandemics. Although select antivirals can be used to treat influenza A virus infection, inherent rapid sequence changes during replication exacerbate the problem of drug resistance, necessitating continuous efforts to develop new drugs. Previous studies have characterized the effects of HSV-1 (ref. 5) and influenza A^18^ infection on host cell glucose metabolism; however, it remains unknown whether these viruses promote increased host cell glutamine consumption or require increased glutamine utilization for optimal replication. We therefore assessed the effect of HSV1 and influenza A infection on host cell glutamine consumption and the impact of GLS inhibition on replication of HSV-1 and influenza A in cultured primary cells.

**Figure 4:**
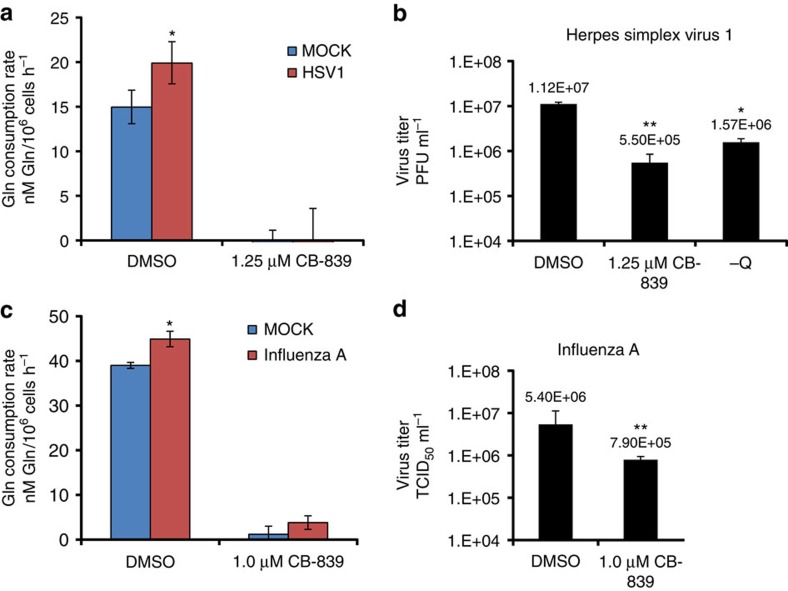
Glutaminase inhibition limits HSV-1 and influenza A replication. (**a**) Glutamine consumption rates of primary human foreskin fibroblasts (HFF) mock infected or infected with HSV-1 (herpes simplex virus 1) at an MOI of 1 for 24 h in the presence of DMSO or 1.25 μM CB-839. (**b**) Infectious viral titer (measured by plaque assay) of supernatant from HFF cells infected with HSV-1 at an MOI of 0.01 for 72 h in the presence of DMSO, 1.25 μM CB839, or in the absence of exogenous glutamine (−Q). (**c**) Glutamine consumption rates of NHBE cells mock infected or infected with influenza A virus at an MOI of 1 for 24 h in the presence of DMSO or 1.0 μM CB-839. (**d**) Infectious viral titer (measured using a TCID_50_ end point dilution assay) of the supernatant from NHBE cells infected with influenza A at an MOI of 1 for 24 h in the presence of DMSO or 1.0 μM CB-839. For (**a**–**d**), error bars denote s.d. (*n*=3), *P<0.05; **P<0.01. Student's *t*-test.

To determine the impact of HSV-1 infection on host cell glutamine consumption, we measured glutamine consumption rate of DMSO-treated HFF 24 h after mock infection or infection with HSV-1 at an MOI of 1. As shown in Fig. 4a, HSV-1 infection significantly increases the glutamine consumption rate of HFF cells. Treatment of HFF cells with 1.25 μM CB-839 dramatically reduces the cellular glutamine consumption rate and prevents the HSV-1-induced increase in glutamine consumption (Fig. 4a). Importantly, treatment of HFF cells with either 1.25 μM CB-839 or glutamine withdrawal significantly reduces HSV-1 replication and lowers viral yields relative to DMSO treatment in the presence of 4 mM glutamine (Fig. 4b). These results suggest that glutamine consumption and GLS activity are important for optimal HSV-1 replication in human foreskin fibroblasts.

To assess the impact of influenza A infection on host cell glutamine consumption, we measured glutamine consumption rate of DMSO-treated primary NHBE cells 24 h after a mock infection or infection with influenza A at an MOI of 1. Influenza A infection significantly increases the glutamine consumption rate of NHBE cells relative to mock-infected cells (Fig. 4c). Treatment of NHBE cells with 1.0 μM CB-839 effectively reduces the glutamine consumption rate and prevents the influenza A-induced increase in cellular glutamine uptake (Fig. 4c). Importantly, similar to results obtained with adenovirus (Fig. 3e) and HSV-1 (Fig. 4b), treatment of NHBE cells with 1.0 μM CB-839 significantly decreases replication of influenza A virus compared with DMSO treatment. These results show that GLS activity is important for optimal influenza A virus replication in primary NHBE cells, and together suggest that pharmacological inhibition of GLS can reduce replication of diverse viruses.

## Discussion

This study demonstrates that MYC-dependent rewiring of host cell glutamine metabolism during adenovirus infection promotes virus replication. These findings are consistent with previous studies showing a critical role for MYC-dependent regulation of glutamine metabolism in growth of cancer cells^8^,^9^,^19^, activated T-lymphocytes^20^, and recently, during latent infection by Kaposi's sarcoma-associated herpesvirus of host endothelial cells^21^. Our data suggests that adenovirus E4ORF1 activation of MYC reprograms host cell glutamine metabolism through transcriptional suppression of miR-23a, upregulation of the glutamine transporters SLC7A5/ASCT2 and SLC1A5/LAT1, and upregulation of enzymes involved in reductive carboxylation and the hexosamine biosynthesis pathway. The specific contribution of each of these components to the altered glutamine metabolism observed during adenovirus infection and viral replication requires further investigation. Importantly, we found that MYC-dependent upregulation of glutamine consumption and GLS activity are necessary for optimal adenovirus replication in primary lung epithelial cells, and that GLS activity is required for optimal replication of HSV-1 and influenza A as well. Whether HSV-1, influenza A, or other viruses also rely on MYC activation to promote reprogramming of glutamine metabolism during infection remains to be determined.

Although previous studies have reported a critical role for increased glutamine consumption and the anaplerotic use of glutamine in host cells infected by HCMV or VACV^7^,^15^, our results tracing the fate of glutamine carbons during adenovirus infection and using a MYC activation-deficient mutant virus have implicated the MYC-directed use of glutamine in additional metabolic pathways, some of which have not been previously characterized in virus-infected cells and are not canonically known to be regulated by MYC. For instance, we detected a MYC activation-dependent increase in reductive glutamine carboxylation in adenovirus-infected cells (Fig. 2b and Supplementary Fig. 2). Consistently, we found MYC activation-dependent upregulation of enzymes involved in reductive carboxylation, including isocitrate dehydrogenase 1 (IDH1), isocitrate dehydrogenase 2 (IDH2), nicotinamide nucleotide transhydrogenase (NNT) and pyruvate dehydrogenase kinase 1 (PDK1) in adenovirus-infected cells (Supplementary Figs 1e and 2). In cancer cells, reductive glutamine metabolism is thought to support growth by providing a source of citrate used for macromolecule synthesis, including for acetyl-CoA production for lipid synthesis in the context of hypoxia^12^,^22^ or mitochondrial dysfunction^13^. The precise functional significance and potential advantages for adenovirus replication provided by enhanced reductive glutamine metabolism remains to be determined. In addition, while HIF has been implicated in regulating reductive glutamine carboxylation in hypoxic cancer cells^23^, our findings are consistent with E4ORF1-induced MYC activation enhancing reductive glutamine carboxylation during adenovirus infection. Whether MYC directly or indirectly modulates reductive carboxylation in other pathophysiological contexts merits further investigation. In addition, since adenovirus infection decreases host cell respiration^6^, it will be interesting to determine whether decreased oxygen consumption is linked to the increased reductive carboxylation observed during adenovirus infection.

Another metabolic pathway found to be altered in a MYC activation-dependent manner during adenovirus infection is the hexosamine biosynthesis pathway (Fig 2e,f, and Supplementary Fig. 3). The hexosamine biosynthesis pathway plays a key role in *N*-linked glycosylation of growth factor receptors to promote their cell surface localization and permit initiation of cell signalling pathways downstream of growth factors^11^,^24^,^25^. Additionally, UDP-GlcNAc generated by the hexosamine biosynthesis pathway is used as the sugar nucleotide donor for *O*-GlcNAc modification of proteins, a highly abundant post-translational modification with emerging roles in regulation of cell signalling and metabolism^26^. For instance, *O*-GlcNAc modification of the glycolytic enzyme, phosphofructokinase 1, increases enzymatic activity, yielding increased glucose flux through the pentose phosphate pathway in cancer cells^27^, and could contribute to the increased glycolytic flux observed in adenovirus-infected cells^6^. Consistent with the increased levels and glutamine carbon labelling of hexosamine biosynthesis pathway intermediates detected in adenovirus-infected cells (Fig. 2e,f), we found that adenovirus infection leads to a MYC-dependent increase in transcript levels of enzymes in the hexosamine biosynthesis pathway, GFAT1 and NAGK (Supplementary Fig. 3). Interestingly, Hepatitis B virus infection also promotes elevated GFAT1 expression and increased hexosamine biosynthesis in hepatoma cells, and GFAT1 inhibition reduces HBV replication^28^. Whether and how GFAT activity and hexosamine biosynthesis contribute to adenovirus replication remains unknown, but our findings support future studies examining potential adenovirus-induced alterations in cellular *N*-linked glycosylation patterns and/or *O*-GlcNAc protein modifications.

In addition to changes in reductive carboxylation and hexosamine biosynthesis, we also detect MYC-dependent changes in amino acid metabolism in adenovirus-infected cells. We found increased conversion of glutamine to aspartate, asparagine and proline post adenovirus infection (Fig. 2c and Supplementary Fig. 2). The increased labelling of aspartate and asparagine from U-^13^C-Gln in AD WT-infected cells is likely due to MYC-dependent routing of glutamine through reductive carboxylation and stimulation of asparagine synthetase activity. The increased labelling of proline from glutamine suggests MYC-induced stimulation of the proline biosynthesis pathway during adenovirus infection. Consistently, in cancer cells MYC promotes enhanced proline biosynthesis from glutamine in part by transcriptional upregulation of pyrroline-5-carboxylate synthase and pyrroline-5-carboxylate reductase 1 (ref. 29), and pyrroline-5carboxylate reductase 1 has been found to be important for optimal growth of breast cancer cell lines^10^. In activated T-cells, MYC promotes increased proline biosynthesis from glutamine to support production of polyamines^20^. Preliminary results suggest increased mRNA expression of proline dehydrogenase (PRODH), an enzyme involved in proline conversion to ornithine, and ornithine decarboxylase, the rate-limiting enzyme of polyamine synthesis in AD WT- versus mock-infected cells (Supplementary Fig. 4). Whether the levels of proline biosynthesis and/or polyamine biosynthesis enzymes are altered in a MYC-dependent manner in adenovirus-infected cells remains to be tested. Interestingly, we also detected a MYC-dependent increase in intracellular amino acid pool sizes along with a MYC-dependent increase in LAT1 and ASCT2 transcript levels during adenovirus infection, consistent with MYC-dependent regulation of use of glutamine for amino acid uptake^3^. The specific contribution of each of these metabolic events to anabolic metabolism during adenovirus infection and viral replication is yet unknown.

Our study shows that CB-839 treatment reduces viral replication of adenovirus, HSV-1, and influenza A in cultured primary cells. This observation highlights the importance of GLS activity in the *in vitro* propagation of a diverse set of viruses and reveals that, in principle, inhibition of glutamine metabolism could be exploited therapeutically to decrease viral replication. While disease caused by adenovirus infection is usually self-limiting and less pathogenic than infection by other viruses, cases of severe adenovirus infections have been rising over the past several decades, specifically in immunocompromised hosts^30^. Limited anti-adenoviral agents are currently available that are efficacious with minimal toxicity. Our proof-of-principle results demonstrate that CB-839-mediated inhibition of GLS activity could potentially be useful to combat adenovirus infection as well as infection by other pathogenic viruses that rely on GLS activity for viral propagation. For HSV-1 and influenza A infections, where antiviral medications are currently available, GLS inhibition by CB-839 may be useful in combination with current standard of care, particularly in resistant cases. However, further studies are needed to determine whether CB-839 can beneficially limit replication of adenovirus, HSV-1 or influenza A *in vivo*. Nonetheless, our results demonstrate the interface between viral and cancer metabolism in upregulation of common metabolic pathways supporting increased proliferation of virions and daughter cells, respectively. Our results suggest that not only can viruses be used to reveal critical targets for cancer metabolism, but that drugs in development to target cancer metabolism may also be useful as antivirals.

## Methods

### Cell protein extract preparation and immunoblot assays

MCF10A cells were obtained from American Type Culture Collection (ATCC) and the laboratory of Dr Frank McCormick (UCSF) and cultured in Dulbecco's modified Eagle's medium and F12 (DMEM:F12) containing 5% Horse serum, 50 U ml^−1^ penicillin-streptomycin, 10 μg ml^−1^ insulin, 0.5 μg ml^−1^ hydrocortisone, 20 ng ml^−1^ EGF, 10 μg ml^−1^ cholera toxin. NBHE cells were obtained from Lonza and cultured in BEGM BulletKit (Lonza). Cells were lysed in either M-PER Mammalian Protein Extraction Reagent (Thermo) with 4 μg ml^−1^ aprotinin, 4 μg ml^−1^ leupeptin, 4 μg ml^−1^ pepstatin, 1 mM PMSF, 1 mM Na3VO4, and 5 mM NaF or NP40 buffer containing 50 mM Tris pH 7.5, 1 mM EDTA, 150 mM NaCl, 1% Nonidet P-40, 4 μg ml^−1^ aprotinin, 4 μg ml^−1^ leupeptin, 4 μg ml^−1^ pepstatin, 1 mM PMSF, 1 mM Na3VO4, and 5 mM NaF. Nuclear and cytoplasmic fractions were prepared using NE-PER extraction reagents (Thermo). Western blot analysis was carried out according to standard methods. Protein concentrations of cell extracts were determined by the Bradford assay. The following commercial antibodies were used as probes: FLAG (Abcam; 1:1,000), GLS (Abcam, AB156876; 1:1,000 for Fig. 1d; Abcam, AB60709; 1:1,000 for Fig. 3b), ASCT2 (Cell Signaling, 1:1,000), MYC (Cell Signaling; 1:1,000), β-Tubulin (Sigma; 1:5,000).

### Viruses

H5wt300 (AD5 WT) was kindly provided by the laboratory of Dr Frank McCormick. AD D68A was derived by subcloning the E4 region into pBluescript (SpeI-BamHI fragment), then performing site-directed mutagenesis, and subsequently recloning back into pAd5. The mutant viruses were propagated on the W162 cell line, which contain and express the E4 region. Infection times of host cells are indicated in the figures at 37 °C with 10 PFU per cell.

### Measurement of glutamine consumption rates

Cellular glutamine consumption rates were measured using a Nova Biomedical Bioprofile Basic Analyzer. Briefly, cells were seeded in 10 cm plates at 70% confluency to ensure the measurements would be taken when the cells were subconfluent. 24 h post seeding, the media was refreshed for all cells, and media was added to 10 cm plate with no cells as a blank control. After 24 h incubation, 1 ml of media was removed from each sample and the blank control, and media samples were analysed in the Nova Bioprofile analyser. Cell number was determined using a Coulter particle analyser.

### Metabolite extraction and metabolomics analysis

Cells were incubated in medium containing 2.5 mM U-^13^C_5_-glutamine for 24 h. The following day, NHBE cells were rinsed with cold 150 mM ammonium acetate (NH4AcO). Cells were carefully scraped off in 800 μl of 50% ice cold methanol. An internal standard of 10 nmol norvaline was added to the cell suspension, followed by 400 μl of cold chloroform. The aqueous layer was transferred to a glass vial and the metabolites dried under vacuum. Metabolites were resuspended in 50 μl 70% ACN and 5 μl of this solution used for the mass spectrometer-based analysis. The analysis was performed on a Q Exactive (Thermo Scientific) coupled to an UltiMate 3000RSLC (Thermo Scientific) UHPLC system. Mobile phase A was 5 mM NH4AcO, pH 9.9, B) was ACN, and the separation achieved on a Luna 3u NH2 100A (150 × 2.0 mm) (Phenomenex) column. The flow was kept at 200 μl min^−1^, and the gradient was from 15% A to 95% A in 18 min, followed by an isocratic step for 9 min and re-equilibration for 7 min. Metabolites we detected and quantified as area under the curve (AUC) based on retention time and accurate mass (≤ 3 p.p.m.) using the TraceFinder 3.1 (Thermo Scientific) software.

Relative amounts of metabolites between various conditions, as well as percentage of labelling were calculated and depicted in bar graphs.

### Quantitative Real-Time PCR

RNA was purified with Qiagen RNeasy Kit. 1 μg of total RNA was used to synthesize cDNA using the iScript cDNA Synthesis Kit (Bio-Rad) as per manufacturer's instructions. Quantitative PCR was conducted on the Roche LightCycler 480 using SYBR Green I Master Mix (Roche) and 0.5 μmol l^−1^ primers. Relative expression values are normalized to control gene (RPL32 or RPLP0) and expressed in terms of linear relative mRNA values. The following primer sequences were used: 5′- AGAATGTACTTGCCAAGGCG -3′ and 5′ CACCATGGTTCTGGTCTCCT -3′ for *Asct2*, 5′- GGCCTTCATCGCAGTACATC -3′ and 5′ ACGCTGTAGCAGTTCACGG -3′ for *Lat1*, 5′- CTTTTGGGTTCCGTCACTTG -3′ and 5′ GTCGTCATGCTTATGGGGAT -3′ for *Idh1*, 5′- TGAACTGCCAGATAATACGGG -3′ and 5′ CTGACAGCCCCCACCTC -3′ for *Idh2*, 5′- CACATTAAGCTGACCAGGCA -3′ and 5′ AGCTCAATACCCCATTGCTG -3′ for *Nnt*, 5′- GGAGGTCTCAACACGAGGTC -3′ and 5′ CGCTGGGTAATGAGGATTTG -3′ for *Pdk1*, and 5′- AGGGGAGGGTTGGAATACAT -3′, 5′ AATGTTTGCCTCTGCCAATC -3′ for *Got2*, 5′- TCGTCTCGTTCGAGGAACAT -3′ and 5′ CACCGAAGCTCGTGTGTG -3′ for *Gfpt1, and* 5′- AGGGGTGGATCCTCTGGTA -3′ and 5′ GGCATCGGTGGTGATTAAGT -3′ for *Nagk.*

### Adenovirus assays

In glutamine withdrawal samples, NHBE cells were plated at subconfluency and media was switched to DMEM/F12 media with or without glutamine/pyruvate 24 h before adenovirus infection. In drug treatment samples, cells were pre-treated with 1.0 μM CB-839 (obtained from Calithera Biosciences, Inc.) or DMSO for 3 h before adenovirus infection. Cells were infected with adenovirus at a MOI of 1 for 72 h. Cells from the entire plate were harvested by pipetting up and down, collected in a conical tube, and spun down at 3,000 r.p.m. for 10 min. The pellet was resuspended with 100 μl 10 mM Tris pH 7.4. Cells were lysed by freezing, thawing and vortexing vigorously. Supernatant was saved as virus stocks for virus titration assays. Infectious virus titer was measured using the 50% Tissue Culture Infective Dose (TCID50). This end point dilution assay quantifies the amount of virus required to produce a cytopathic effect in 50% of inoculated tissue culture cells. In 96 well plates, HeLa cells were plated at 2,500 cells per well and subsequently infected with 10-fold dilutions of virus stocks. Dilutions of virus stocks were added to cells in replicates of 10, and cytopathic effect was manually observed and recorded for each virus dilution 5 days post infection. Results were used to mathematically calculate a TCID_50_ result.

### Herpes simplex 1 assays

HSV-1 strain 17+ stock was generated by infecting Vero cells at an MOI of 0.05 PFU per cell. Human foreskin fibroblast cells and Vero cells were cultured in complete Dulbecco modification of Eagle medium (DMEM) containing 10% fetal bovine serum, supplemented with penicillin and streptomycin. HFF cells were pre-treated with 1.25 μM CB-839 for 3 h, and then infected with HSV-1 virus at an MOI of 0.01 with or without the presence of 1.25 μM CB839 or, in the case for glutamine withdrawal, DMEM media without L-glutamine and without pyruvate. Supernatant containing the virus was harvested 72 h post infection and titred by plaque assay. Plaque assay was performed in monolayers of Vero cells overlaid with 1% methylcellulose.

### Influenza A assays

NHBE cells were plated at subconfluency. Cells were pre-treated with 1.0 μM CB-839 for 3 h or DMSO control, and then infected with influenza virus (H1N1/WSN/33) at an MOI of 1 with or without the presence of 1.0 μM CB-839. Supernatant containing the virus was collected 24 h post infection and TCID50 were assayed with A549 cells.

### Micro RNA analysis

Micro RNAs were quantified using the qScript microRNA cDNA synthesis kit (Quanta Biosciences). Briefly, enriched miRNA was prepared from 100 mm plates of subconfluent NHBE cells mock infected, or infected with Ad WT or Ad D68A at the indicated times using Qiagen miRNeasy Mini Kit (Qiagen). miRNAs were first polyadenylated in a poly(A) polymerase reaction, and then reverse transcribed into cDNA with oligo-dT adapter primers using the first-strand cDNA synthesis reaction mixture. Real-time SYBR Green qRT-PCR was performed using 200 nM of each PerfeCTA microRNA Assay primer (hsa-miR23a-3p and hsamiR23b-3p) and PerfeCTA Universal Primer. Quantitative PCR was conducted on the Roche LightCycler 480. Relative values were normalized against the small nucleolar RNA SNORD43, and values were compared against mock-infected samples.

### Statistical analysis

All data are presented as ±s.d. As noted in figure legends, data were analysed by two-sample equal variance Student's *t*-test with two-tailed analysis.

## Additional information

**How to cite this article:** Thai, M. *et al*. MYC-induced reprogramming of glutamine catabolism supports optimal virus replication. *Nat. Commun.* 6:8873 doi: 10.1038/ncomms9873 (2015).

## Supplementary Material

Supplementary Information Supplementary Figures 1-5
