# Supplementary material for: MYC-induced reprogramming of glutamine catabolism supports optimal virus replication
Source: Nat Commun. 2015 Nov 12;6:8873. doi: 10.1038/ncomms9873 (PMC4660206; doi:10.1038/ncomms9873)
Supplement: Supplementary Information — Supplementary Figures 1-5 [file ncomms9873-s1.pdf]

Supplementary Figures

Supplementary Figure 1 (Christofk)

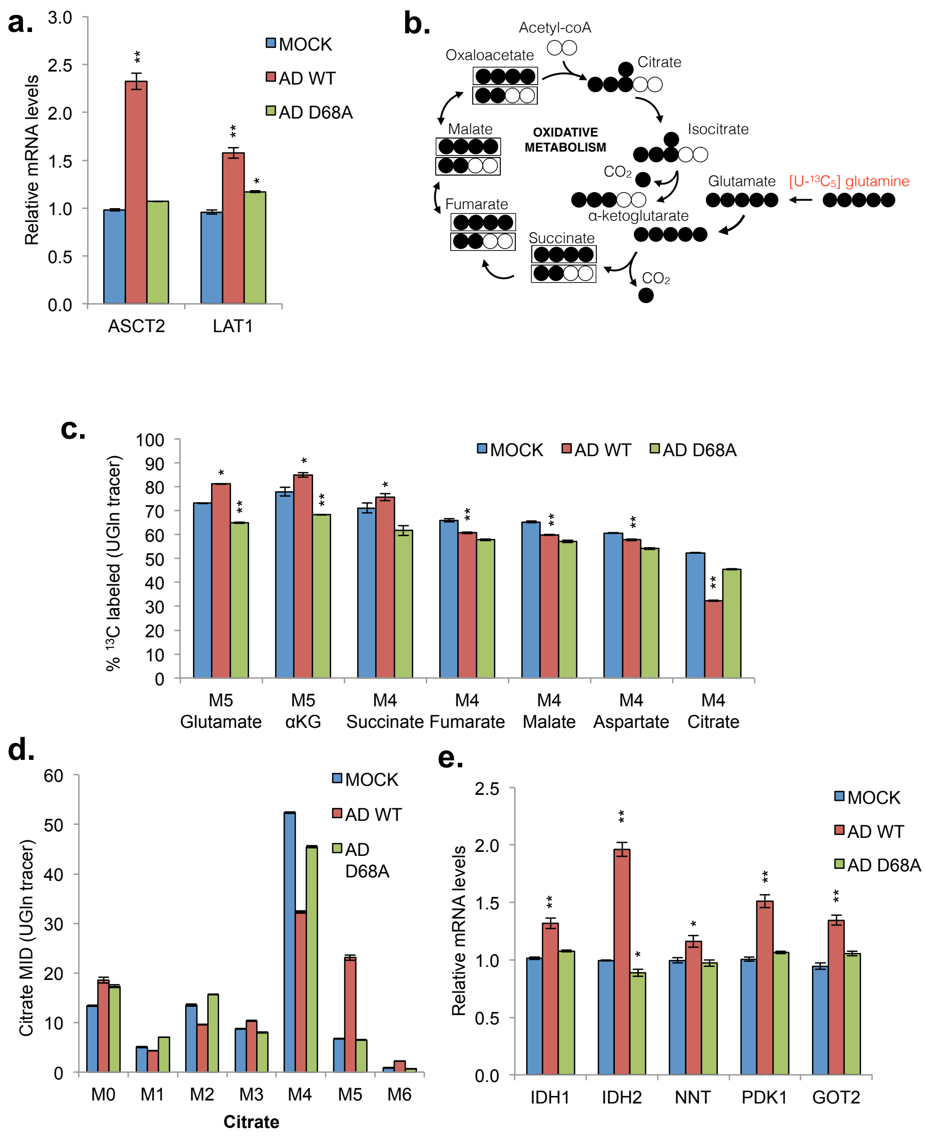

Supplementary Figure 1:
(a) qRT-PCR was performed on RNA collected from MCF10A cells 8 hours post infection with AD WT or AD ORF1 D68A virus. Relative levels of ASCT2 and LAT1 are shown. For (b)-(e), NHBE cells were labeled with U-^{13}C-glutamine (UGln tracer) and mock infected or infected with AD WT or AD ORF1 D68A at an MOI of 1. 24 hours post infection, intracellular metabolites were extracted and analyzed by LC-MS/MS. (b) Schematic tracing the fate of ^{13}C atoms from U-$^{13}C_5$-glutamine in oxidative metabolism in the TCA cycle. (c) Percentage of ^{13}C-labeled isotopomers of intermediates in oxidative TCA cycle metabolism. (d) Citrate mass isotopomer distribution (MID) resulting from U-^{13}C-glutamine labeling. (e) qRT-PCR was performed as described in (a), and relative levels of transcripts involved in reductive carboxylation are shown.

Supplementary Figure 2 (Christofk)

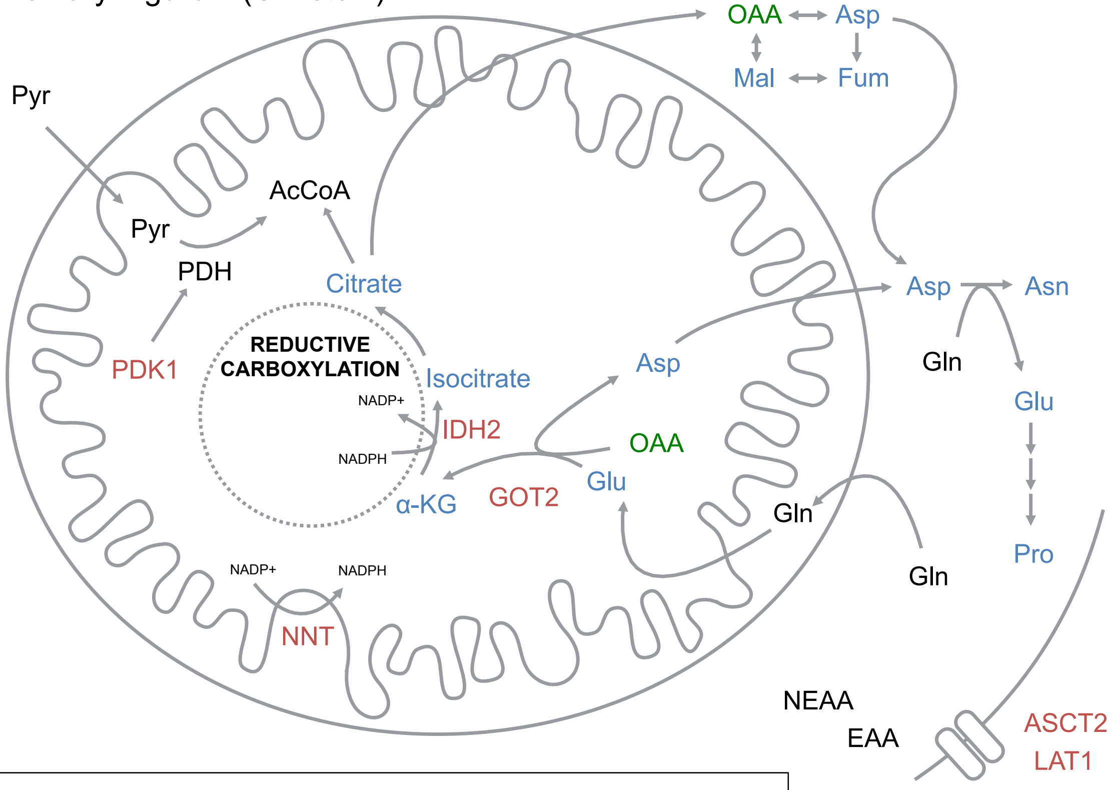

KEY:
Metabolite increased labeling from U-13C-Gln in AD WT but not AD ORF1 D68A infected cells
Metabolite difficult to measure via current LC-MS method
Enzyme transcript level elevated in AD WT but not AD ORF1 D68A infected cells

Supplementary Figure 2:
Schematic of reductive glutamine carboxylation and glutamine-associated amino acid metabolism events summarizing the MYC-dependent changes observed in adenovirus-infected cells.

Supplementary Figure 3 (Christofk)

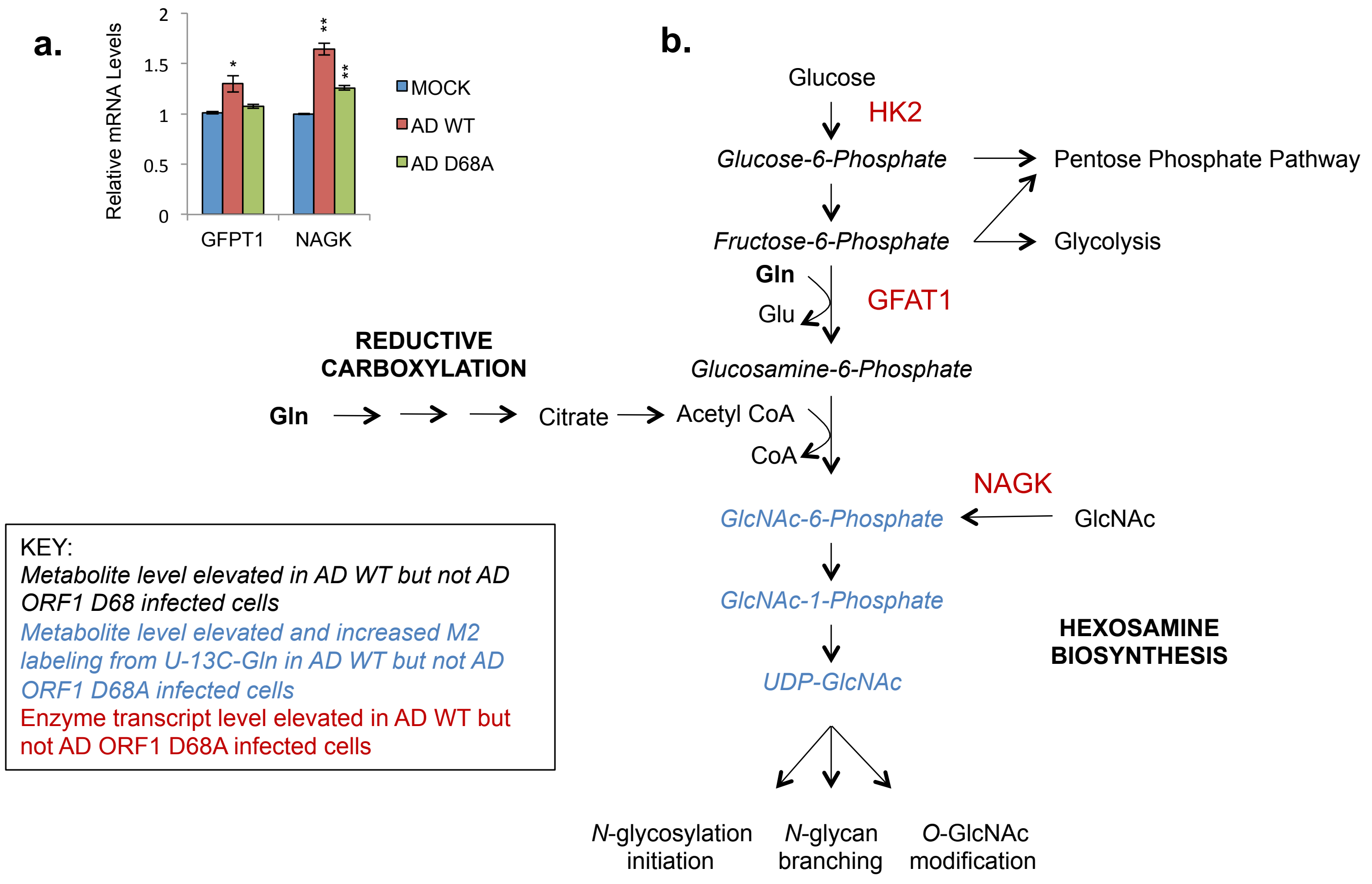

Supplementary Figure 3:
(a) qRT-PCR was performed on RNA collected from MCF10A cells 8 hours post infection with AD WT or AD ORF1 D68A virus. Relative levels of GFPT1 (glutamine fructose-6-phosphate amidotransferase 1) and NAGK (N-acetylglucosamine kinase) are shown. (b) Schematic of the hexosamine biosynthesis pathway summarizing the MYC-dependent changes observed in adenovirus-infected cells.

Supplementary Figure 4 (Christofk)

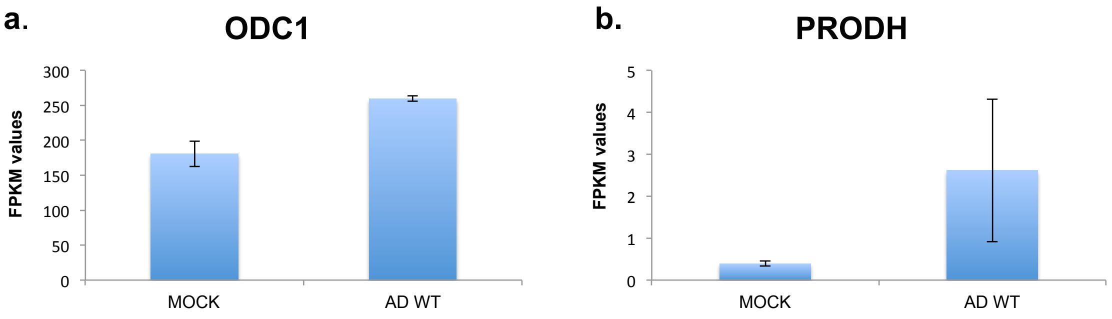

Supplementary Figure 4:

Preliminary RNA-sequencing data comparing uninfected NHBE cells (MOCK) versus cells infected for 8 hours with AD WT (AD WT). The fragments per kilobase of transcript per million mapped (FPKM) values indicate that RNA levels of (a) ornithine decarboxylase (ODC1) and (b) proline dehydrogenase (PRODH) are increased at 8 hours post-AD WT infection.

Supplementary Figure 5 (Christofk)

a.

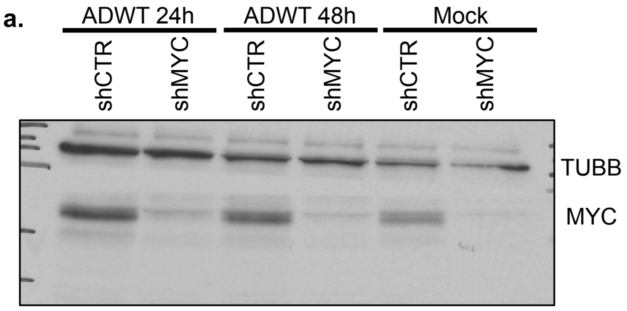

Figure 1b

b.

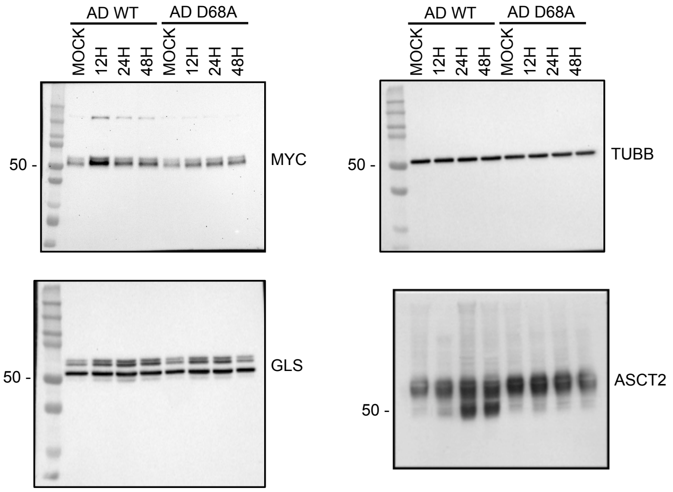

Figure 1d

c.

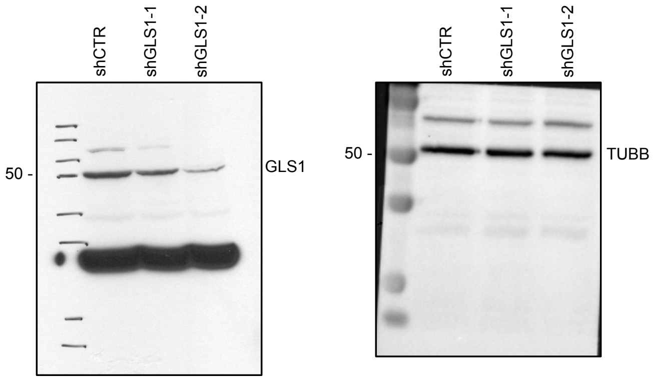

Figure 3b

Supplementary Figure 5:
Uncropped Western Blot images presented in this manuscript. Figure labeling corresponds to the figure in the main manuscript.
